# From Staple Removal to Startle—The Unexpected Discovery of a Scalp Pseudoaneurysm: A Case Report

**DOI:** 10.5811/cpcem.63965

**Published:** 2026-09-20

**Authors:** Brendon Stankard, John Forrester, Stephanie Jose, Allison Cohen, Mathew Nelson

**Affiliations:** North Shore University Hospital, Department of Emergency Medicine, Manhasset, New York

**Keywords:** pseudoaneurysm, point-of-care ultrasound, ultrasound, scalp trauma, doppler

## Abstract

**Introduction:**

Point-of-care ultrasound (POCUS) allows emergency physicians to rapidly diagnose vascular abnormalities, including pseudoaneurysms. Although commonly identified following vascular catheterization, traumatic pseudoaneurysms of superficial scalp arteries are rare.

**Case Report:**

An 85-year-old woman receiving anticoagulation presented to the emergency department (ED) for removal of a scalp staple placed during treatment of a previously bleeding scalp wound. Physical examination revealed a persistent scalp hematoma. Point-of-care ultrasound demonstrated a hypoechoic collection with Doppler flow consistent with a pseudoaneurysm. Computed tomography angiography confirmed the diagnosis. Ultrasound-guided compression attempted in the ED resulted only in transient thrombosis. The patient subsequently underwent elective percutaneous embolization.

**Conclusion:**

Emergency physician–performed POCUS can rapidly identify traumatic scalp pseudoaneurysms and facilitate timely consultation and treatment.

## INTRODUCTION

Point-of-care ultrasound (POCUS) has become an important diagnostic tool in emergency medicine for the rapid identification of vascular abnormalities. Pseudoaneurysms most commonly occur following vascular catheterization, particularly involving the femoral artery, and are frequently diagnosed using ultrasound in the emergency department.^3^ Traumatic pseudoaneurysms of superficial scalp arteries are uncommon but represent an important consideration in patients presenting with persistent swelling following head trauma. We report a case of a superficial scalp pseudoaneurysm discovered incidentally during staple removal and diagnosed with POCUS in the emergency department (ED).

## CASE REPORT

An 85-year-old woman with a past medical history of hypertension, hyperlipidemia, type 2 diabetes mellitus, and atrial fibrillation treated with apixaban presented to the ED for removal of a scalp staple placed one week earlier. Approximately one month prior, the patient had fallen down three stairs and sustained a head injury. She was initially evaluated in the ED and found to have an abrasion over the right superior occiput without active bleeding and a small underlying scalp hematoma on noncontrast computed tomography (CT). She was discharged home without intervention.

Two weeks later, she returned to the ED after developing bleeding from the area of the hematoma. Repeat noncontrast CT again demonstrated a small scalp hematoma. One staple was placed to control bleeding, and the patient was discharged.

At the most recent visit for staple removal, the patient’s vital signs were notable for the following: blood pressure, 179/68 mm Hg; heart rate, 60 beats per minute; respiratory rate, 20 breaths per minute; temperature, 97.8 °F orally; and oxygen saturation, 98% on room air. The wound appeared completely closed without active bleeding. However, a persistent scalp hematoma was noted on examination. The hematoma was around 3 cm, dome-shaped, and indurated. There was no active bleeding from it at that time. There was no thrill, and the area was slightly tender to palpation. Given this finding and the persistence of a hematoma that would have been expected to be resolving by that point, POCUS was performed to assess for underlying vascular abnormality, including possible pseudoaneurysm formation.

Initial ultrasound demonstrated a hypoechoic fluid collection measuring 2.9 × 1.5 × 2.9 cm (Image 1). Color Doppler imaging demonstrated internal blood flow consistent with a 1.4 × 0.7 × 1.0 cm pseudoaneurysm (Image 2). Computed tomography angiography of the head and neck confirmed a faint blush suggestive of pseudoaneurysm formation (Image 3).

Neurosurgery consult recommended ultrasound-guided manual compression in the ED. Compression produced transient thrombosis but did not achieve durable occlusion. The patient was discharged with follow-up and subsequently underwent elective direct percutaneous embolization of the occipital pseudoaneurysm.

## DISCUSSION

Pseudoaneurysms develop when disruption of an arterial wall allows blood to escape into surrounding tissues while maintaining communication with the parent vessel. Persistent arterial pressure leads to formation of a fibrous sac containing circulating blood that communicates with the arterial lumen.^1^

Pseudoaneurysms may result from blunt or penetrating trauma, inflammatory processes, infection, or iatrogenic arterial injury during medical procedures.^1^ Vascular catheterization is a common cause, with reported incidence rates approaching 7.7%.^1^ Although rare, persistent posttraumatic scalp hematoma may indicate underlying pseudoaneurysm formation, with trauma representing the most common cause.^6^

*CPC-EM Capsule*

What do we already know about this clinical entity?

*Scalp pseudoaneurysms are rare delayed complications of blunt trauma and can mimic benign scalp hematomas or soft tissue swelling.*

What makes this presentation of disease reportable?

*The pseudoaneurysm was discovered incidentally during routine staple removal, with point-of-care ultrasound (POCUS) expediting diagnosis before potentially harmful treatment.*

What is the major learning point?

*POCUS should be considered for persistent or pulsatile scalp masses after trauma to rapidly identify pseudoaneurysm and avoid inappropriate procedures.*

How might this improve emergency medicine practice?

*Routine use of POCUS for atypical post-traumatic scalp lesions can prevent misdiagnosis, guide management, and expedite definitive treatment.*

Scalp pseudoaneurysms should be considered in patients presenting with persistent or enlarging posttraumatic scalp hematomas that are not resolving as expected.^6^,^7^ Although pulsatility, bruit, and thrills may be present on physical exam, they are not always noted.^6^ In such cases, POCUS provides a rapid, safe, and easily accessible modality for identifying underlying vascular lesions.^6^ Point-of-care ultrasound provides a rapid and accessible method for diagnosing pseudoaneurysms in the ED.^6^ On B-mode ultrasound, pseudoaneurysms appear as an anechoic or hypoechoic sac adjacent to the affected artery.^1^

Color Doppler imaging typically demonstrates the classic “yin–yang” sign, reflecting swirling bidirectional blood flow within the pseudoaneurysm cavity.^2^ The diagnostic hallmark of pseudoaneurysm is identification of a communicating neck between the pseudoaneurysm sac and the feeding artery with a “to-and-fro” Doppler waveform, representing bidirectional blood flow entering and exiting the sac during the cardiac cycle.^1^,^2^

Ultrasound-guided compression can be used as an initial treatment approach for pseudoaneurysms. Direct pressure is applied with the ultrasound transducer to occlude flow at the pseudoaneurysm neck while preserving distal arterial perfusion. Sustained compression for approximately 10 minutes may promote thrombosis.^3^ However, the success of compression therapy may be limited by patient discomfort, operator fatigue, lesion size or depth, and anticoagulation therapy.^1^ In such cases, alternative treatments such as thrombin injection or endovascular embolization may be necessary.

## CONCLUSION

Persistent hematomas following trauma should prompt consideration of pseudoaneurysm formation. Emergency physician–performed POCUS can rapidly identify superficial vascular injuries and guide timely consultation and treatment.

## Figures and Tables

**Image 1:**
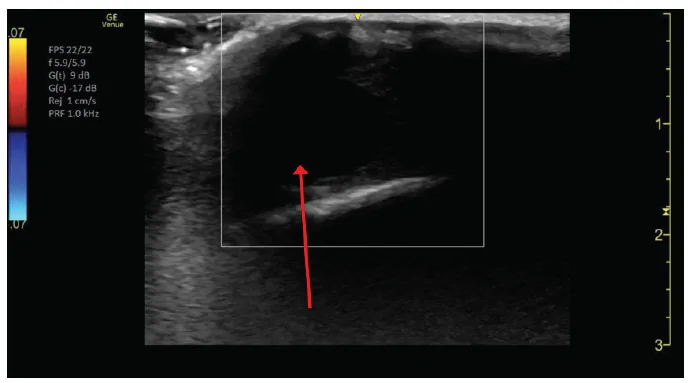
B-mode ultrasound demonstrating a hypoechoic fluid collection within the scalp measuring 2.9 × 1.5 × 2.9 centimeter.

**Image 2:**
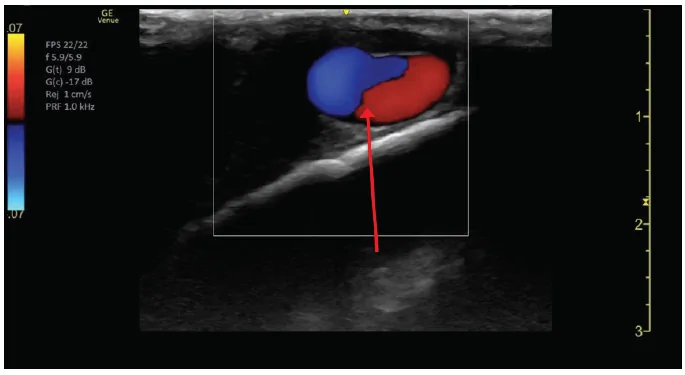
Color Doppler ultrasound demonstrating internal swirling blood flow within the lesion consistent with pseudoaneurysm (“yin–yang sign”).

**Image 3:**
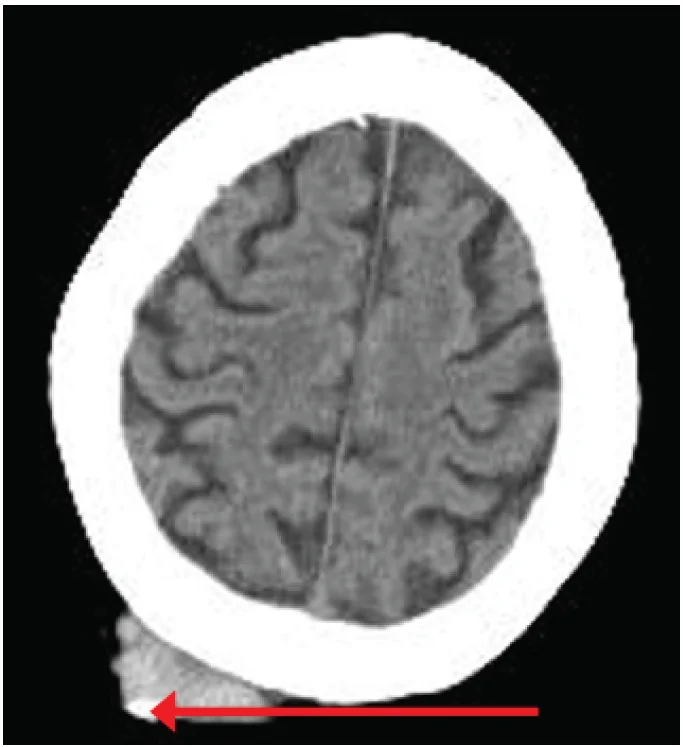
Computed tomography angiogram of the head and neck demonstrating a hematoma with a faint blush measuring 1.4 × 0.7 × 1 centimeter.
